# Transcatheter embolization with Squid, combined with other embolic agents or alone, in different abdominal diseases: a single-center experience in 30 patients

**DOI:** 10.1186/s42155-019-0051-7

**Published:** 2019-02-04

**Authors:** Massimo Venturini, Carolina Lanza, Paolo Marra, Anna Colarieti, Marta Panzeri, Luigi Augello, Simone Gusmini, Marco Salvioni, Francesco De Cobelli, Alessandro Del Maschio

**Affiliations:** 1 Department of Radiology, Scientific Institute H S. Raffaele, Via Olgettina 60, 20132 Milan, Italy; 2 Vita-Salute San Raffaele University, Milan, Italy

**Keywords:** Transcatheter embolization, Squid, Portal vein embolization, Visceral aneurysm, Arteriovenous malformation, Type 2 endoleak

## Abstract

**Background:**

Squid, as Onyx, is an ethylene-vinyl alcohol copolymer (EVOH)-based liquid embolic agent developed for neuroradiologic interventions with poor application in abdominal district. Our aim was to evaluate safety, complications, and efficacy of transcatheter embolization using the two available formulations Squid-18 and 12, in 30 patients affected by different abdominal diseases.

**Results:**

Transcatheter embolization with Squid, combined with other embolic agents, as poly vinyl alcohol (PVA) particles, coils and amplatzer plugs, or alone (type 2 endoleak), was performed in 30 patients, as follows: 10 portal vein embolizations (PVEs), 6 arteriovenous malformations (AVMs), 5 visceral artery aneurysms (VAAs), 4 type 2 endoleaks, 3 preoperative embolizations, 1 acute arterial bleeding, 1 female varicocele. Squid was always administered using dimethyl sulfoxide (DMSO) compatible microcatheters. Technical success, 30-day clinical success and complications were assessed.

Technical success was 90%. 3 patients (2 AVMs, 1 VAA) required re-intervention successfully performed in all cases. Major complications, cases of microcatheter entrapment and DMSO-related poor pain control were not recorded. 30-day clinical success was 93.3%: in 2 patients submitted to PVE a sufficient future liver remnant (FLR) hypertrophy was not achieved.

**Conclusion:**

Squid was successfully used with low complication rate in many abdominal diseases showing a valid embolic action either combined with other embolic agents or alone in type 2 endoleak. The availability of different formulations (Squid-18 and Squid-12) variable for viscosity makes Squid preferable to Onyx as EVOH-based liquid embolic agent, even though comparable studies in different abdominal districts with a larger cohort of patients will be necessary.

## Introduction

Transcatheter embolization (TE) is a well-established technique in interventional radiology in which an occlusive agent is delivered through a catheter to obstruct flow within a targeted blood vessel (Sheth et al., 2017). Each embolic agent is characterized by its respective strengths and weaknesses and can be used alone or combined with other occlusive agents to enhance its embolic power. Embolic agents can also divided into solids, as coils for example, and liquids (Brassel & Meila, 2015): liquid embolic materials include adhesive agents, like acrylates, like n-butyl cyanoacrylate (n-BCA) (Kim et al., 2017), non-adhesive agents, such as ethylene-vinyl alcohol copolymer (EVOH) (Sadeh-Gonike et al., 2018) and cytotoxics, like ethanol (Sakuhara et al., 2015). Coils are largely used in TE: advantages of coils are their marked radiopacity and ability of replacement or removal if necessary but their major limit is the difficulty to obtain a complete occlusion. The advantage of an adhesive liquid embolic agent as n-BCA is a quick induction of a permanent thrombosis after polymerization. Using non adhesive liquid embolic agents composed of EVOH copolymer dissolved in dimethyl sulfoxide (DMSO) and mixed with micronized tantalum powder, as Onyx, it is possible to obtain a slower solidification, a more prolonged injection time and a reduced risk of microcatheter entrapment than n-BCA. Onyx, first described in 1990s, represents the well-known liquid embolic agent widely used in cerebral arteriovenous malformations (AVMs) (Gemmete et al., 2009; Singfer et al., 2017; Alturki et al., 2018; Nerva et al., 2018) and also in abdominal-peripheral applications (Cobb et al., 2014; Né et al., 2018; Izaaryene et al., 2016; Regine et al., 2015; Chevallier et al., 2016), particularly in the management of endoleak post Endovascular Aortic Repair (EVAR) (Marcelin et al., 2017; Ierardi et al., 2018). Squid is a new DMSO- and EVOH-based liquid embolic agent which has mainly been used in cerebral field (Akmangit et al., 2017; Gioppo et al., 2017) with poor application in abdominal district (Szatmáry et al., 2017). It is commercially available in Squid-12 (Szatmáry et al., 2017; Erbahceci et al., 2014) and Squid-18 forms. Squid-12, due to its lower viscosity with more distal penetration capability, may have additional advantage in AVMs embolization over Onyx, unavailable in this formulation. The aim of the present study was to report safety, complications, and efficacy of transcatheter embolization using Squid-18 and Squid-12, combined with other embolic agents or alone, in 30 patients affected by different abdominal diseases.

## Materials and methods

### Study design

A retrospective review of a prospectively maintained database was performed to collect data about TE procedures in the abdominal district using Squid-Peri (Emboflu, Gland, Switzerland) from November 2016 to May 2018. Squid® in both formulations 18 and 12 was approved in Europe (CE marking for medical devices) for embolization of lesions in the peripheral vasculature, including arteriovenous malformations and hypervascular tumors. Squid-18 and Squid-12, variable for viscosity, were both used as liquid embolic agent, based on ethylene vinyl alcohol copolymer (EVOH) after intra-arterial infusion of the solvent dimethyl sulfoxide (DMSO). Squid was deliberately used combined with other embolic agents or alone in different abdominal diseases requiring TE. The indication to use Squid, similarly to Onyx (Medtronic, Dublin, Ireland), rather than other embolic agents was deemed necessary to achieve a more rapid and permanent vessel occlusion.

Written informed consent was obtained from all patients. Institutional review board approval was not required because of the retrospective nature of the work.

Technical success, peri-procedural complications, 30-day clinical success (imaging/clinical data at 1 month), and post-procedural complications were assessed according to international standard guidelines (Angle et al., 2010): technical success was defined as an immediate angiographic result of complete occlusion of target vessel(s); secondary technical success was considered in case of complete occlusion of target vessel(s) after re-intervention; 30-day clinical success was based on clinical improvements of signs and symptoms and imaging data. About complications, minor complications were defined as complications without sequelae or requiring nominal therapy or short hospital stay for observation (generally overnight); major complications were defined as: (1) complications that require therapy and minor hospitalization (< 48 h); (2) complications that require major therapy, unplanned increase in level of care, prolonged hospitalization (> 48 h); (3) complications that require permanent adverse sequelae; (4) death. Patients requiring re-intervention with a further embolization were also assessed. Embolization procedures performed in election or in emergency were distinguished. Cases of microcatheter entrapment or poor pain control during DMSO infusion were recorded.

### Patient selection criteria: Clinical and imaging indications

Patient selection criteria were based on clinical and imaging data. Imaging was based on a triphasic multidetector contrast-enhanced Computed Tomography (CT) scan (Brilliance 64, Philips Healthcare, Cleveland OH, USA) performed before embolization procedure and 1 month later, as gold standard technique, even though in particular cases Magnetic Resonance Imaging (MRI) or Color Doppler Ultrasound (CDU) were also used. Indications to transcatheter embolization were shared by clinicians, surgeons and interventional radiologists according to different selection criteria, tailored to the specific abdominal diseases and according to standard guidelines. Pre-operative portal vein embolization (PVE) was performed in patients affected by extended malignant liver tumors, candidates to right hepatectomy, to increase the volumetric ratio of future liver remnant (FLR) to total liver volume, in case of insufficient volume of the left hepatic lobe (Shinkawa et al., 2017; van Lienden et al., 2013). Visceral artery aneurysms (VAAs) were embolized in case of diameter larger than 2 cm or showing progressive enlargement over time (Venturini et al., 2017; Venturini et al., 2018). Abdominal arterio-venous malformations (AVMs) were treated in symptomatic patients (Lv et al., 2017; Yoon et al., 2016). Type 2 endoleak embolization was performed in patients showing progressive enlargement of the aneurysmatic sac post EVAR with a suspected type 2 endoleak at CT-angiography (Rahimi et al., 2018; Ribé et al., 2017). Pre-surgical arterial embolization was performed in case of voluminous masses or hypervascular tumors (Lau et al., 2013). Varicocele embolization was performed in case of chronic pelvic pain (Laborda et al., 2013). Transcatheter embolization with Squid of an acute bleeding was performed in case of a large arterial breach with significant extravasation of contrast. The final decision to use Squid, combined with other embolic agents (or alone), was taken by the interventional radiologist based on the angiographic evidence and the specific scenario. Squid-18 was used to obtain a permanent plug adjacent to the microcatheter tip for example in TE of VAAs, while Squid-12 was used to obtain a more distal penetration for example in PVE.

### Transcatheter embolization

All procedures of transcatheter embolization were performed in an angiographic room by interventional radiologists with at least 10 year of experience in the field, using digital subtraction angiography (DSA) (Philips Allura Xper FD20, Angio system, Netherlands). Procedures technically varied according to different type of transcatheter embolization. Squid was always administered after a super-selective catheterization with a DMSO-compatible microcatheter.

### PVE patients subgroup

Right PVE was performed in patients with extensive malignant hepatobiliary disease due to hepatocellular carcinoma (HCC), cholangiocarcinoma or colorectal liver metastases, involving the right hepatic lobe, with insufficient left hepatic lobe and candidates to right hepatectomy (or right trisectionectomy). Contrast-enhanced CT was performed before the procedure and 4 weeks after right PVE to assess future liver remnant (FLR) hypertrophy. Pre-procedural data, such as future liver remnant volume (FLR-V), total estimated liver volume (TELV), embolized liver volume, the ratio FLRV/TELV and the same post-procedural data, were collected. For 7 patients, dynamic 99mTc-mebrofenin hepatobiliary scintigraphy (HBS) was performed, in order to define an accurate pre-operative risk analysis (Olthof et al., 2017), evaluating the total liver-function (TL-F) corrected for body surface area (BSA) and expressed as %/min/m2 (cut off 2.69%/min/m2). Technical success was defined as complete occlusion of portal branches of hepatic lobes to be resected. Clinical success was defined as the achievement of volumetric criteria necessary for hepatic resection: an increase of Future FLR-V above the cut off of 25 of total preoperative liver volume was considered sufficient for a safe resection in patients with normal liver function; whereas in patient with compromised liver (e.g. cirrhosis, previous chemotherapy) a % of FRL-V above the cut off of 40% was deemed necessary (van Lienden et al., 2013).

### Statistical analysis

Descriptive analysis were performed on the dataset and presented in simple frequencies, proportion and percentages using Microsoft Excel 2011 (Microsoft Corporation, Redmond, WA, USA) from November 2016. *P*-values, calculated by T student test, were considered significant for values < 0.05. The statistical analyses, the Kaplan-Meier survival curves and the graph were performed using Graph-Pad Prism software (Version 6; GraphPad, Inc., San Diego, CA).

## Results

### General findings

**Fig. 1:**
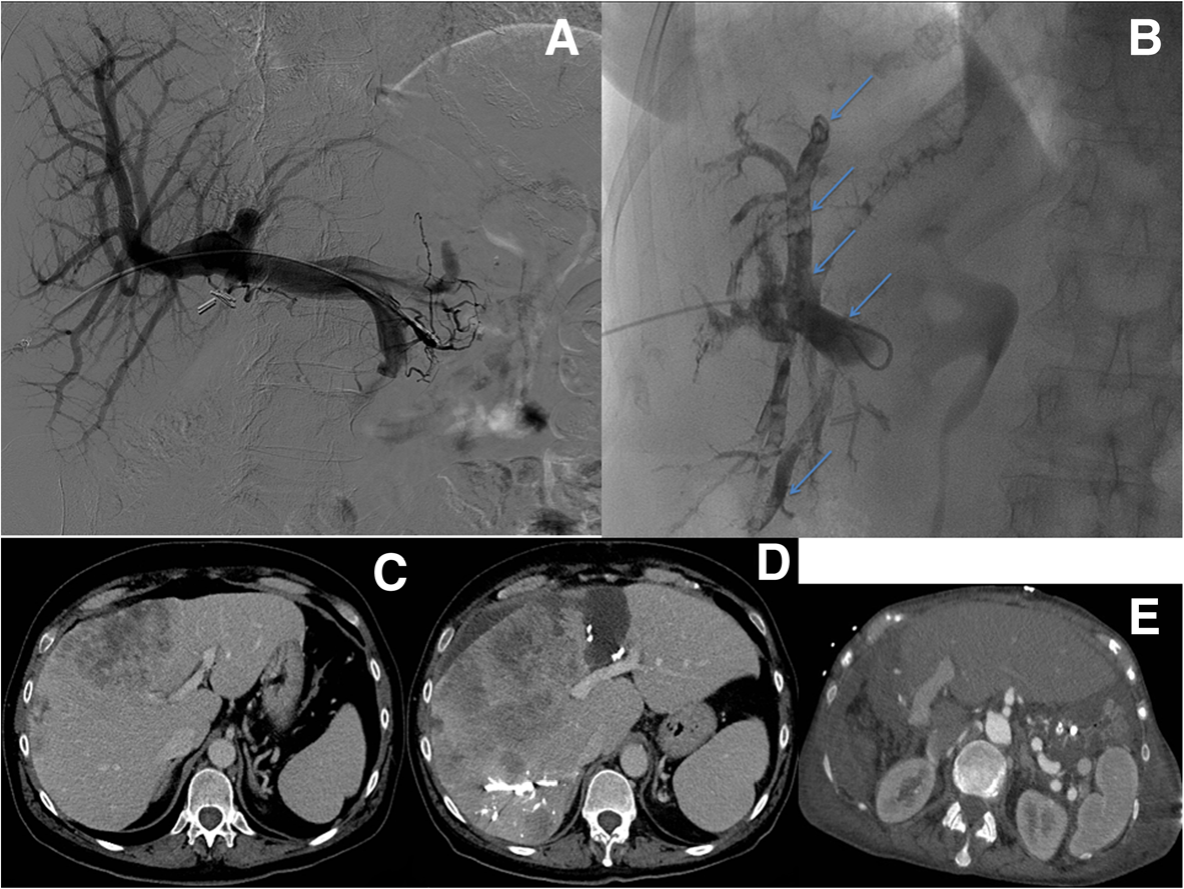
Portal Vein Embolization (PVE) before right hepatectomy in a patient affected by Cholangiocarcinoma. **a** Preliminary portography before right PVE. **b** Right PVE using PVA particles and Squid-12 (light blue arrows). **c** Contrast enhanced CT pre-PVE showing an insufficient volume of left hepatic lobe. **d** Contrast enhanced CT post-PVE showing an increase of left hepatic lobe, with an increase of FLR-V of 71%. **e** Contrast enhanced CT post- right hepatectomy shows hypertrophy of the left hepatic lobe (FLR)

**Fig. 2:**
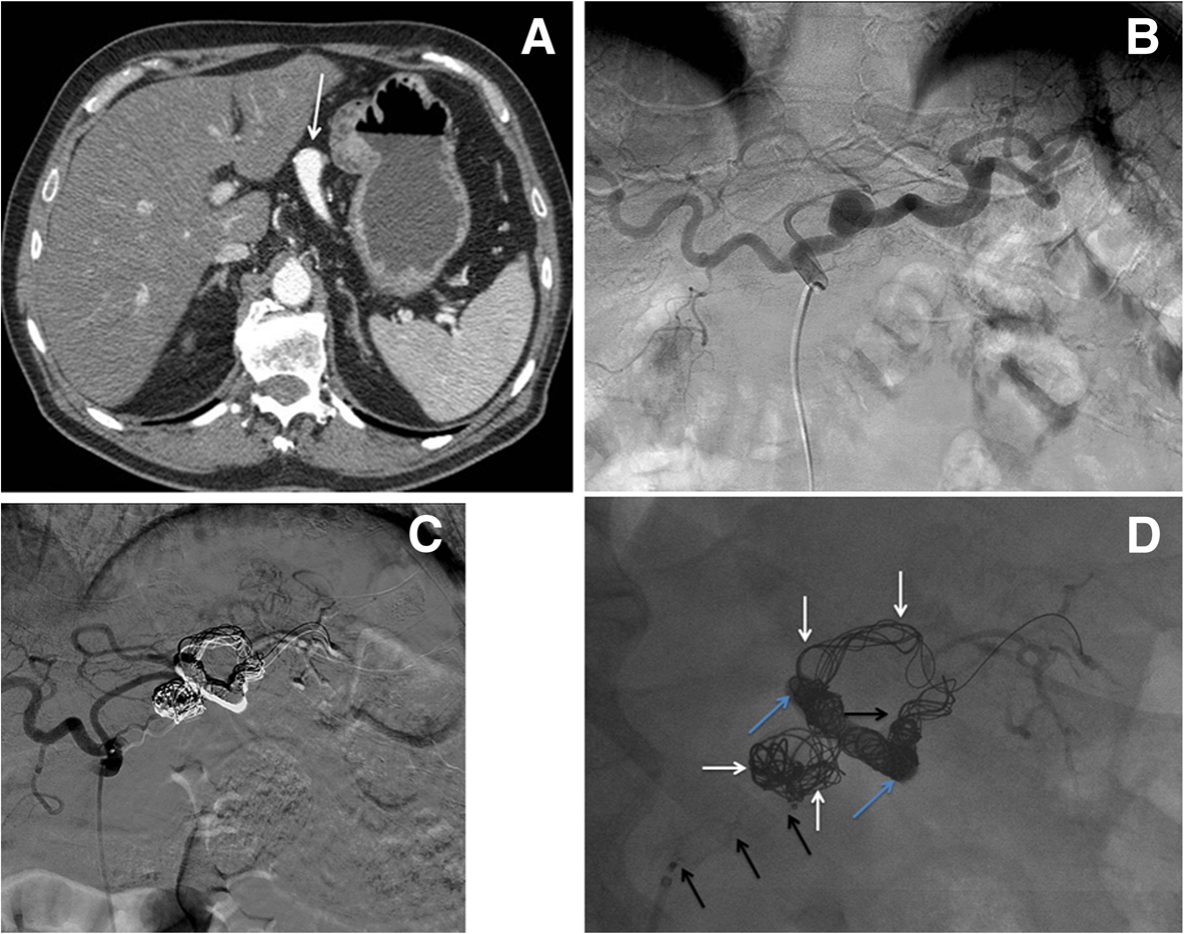
Visceral artery aneurysm (VAA) embolization. **a** Contrast-enhanced CT in arterial phase shows a splenic artery aneurysm (white arrow). **b** Transfemoral diagnostic angiography of the celiac trunk confirms the presence of the splenic artery aneurysm. **c** After VAA embolization, DSA shows aneurysm exclusion, splenic artery occlusion with preserved patency of its distal branches through gastric collaterals. **d** X-rays show embolic agents used for splenic artery aneurysm embolization: Squid (light blue arrows), coils (white arrows) and Amplatzer plug (black arrows)

**Fig. 3:**
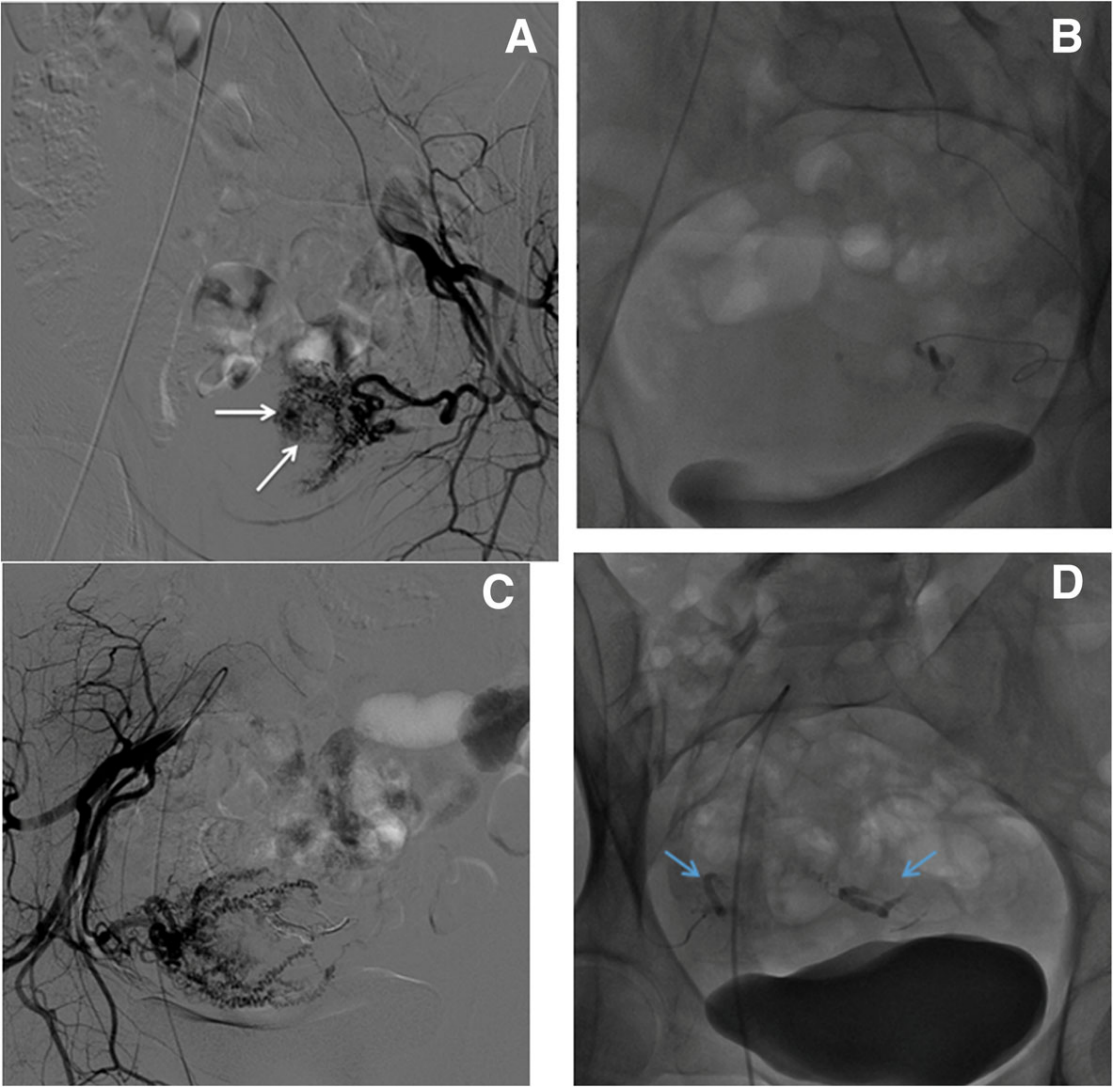
Arteriovenous Malformation (AVM) embolization**. a** Right transfemoral DSA of the left hypogastric artery shows an uterine AVM (white arrows). **b** A superselective embolization with microcatheter of the left uterine artery is performed using PVA particles and Squid-18. **c** Diagnostic DSA of the right hypogastric artery. **d** After bilateral uterine AVM embolization, X-rays show both uterine arteries opacified with Squid (light blue arrows)

**Fig. 4:**
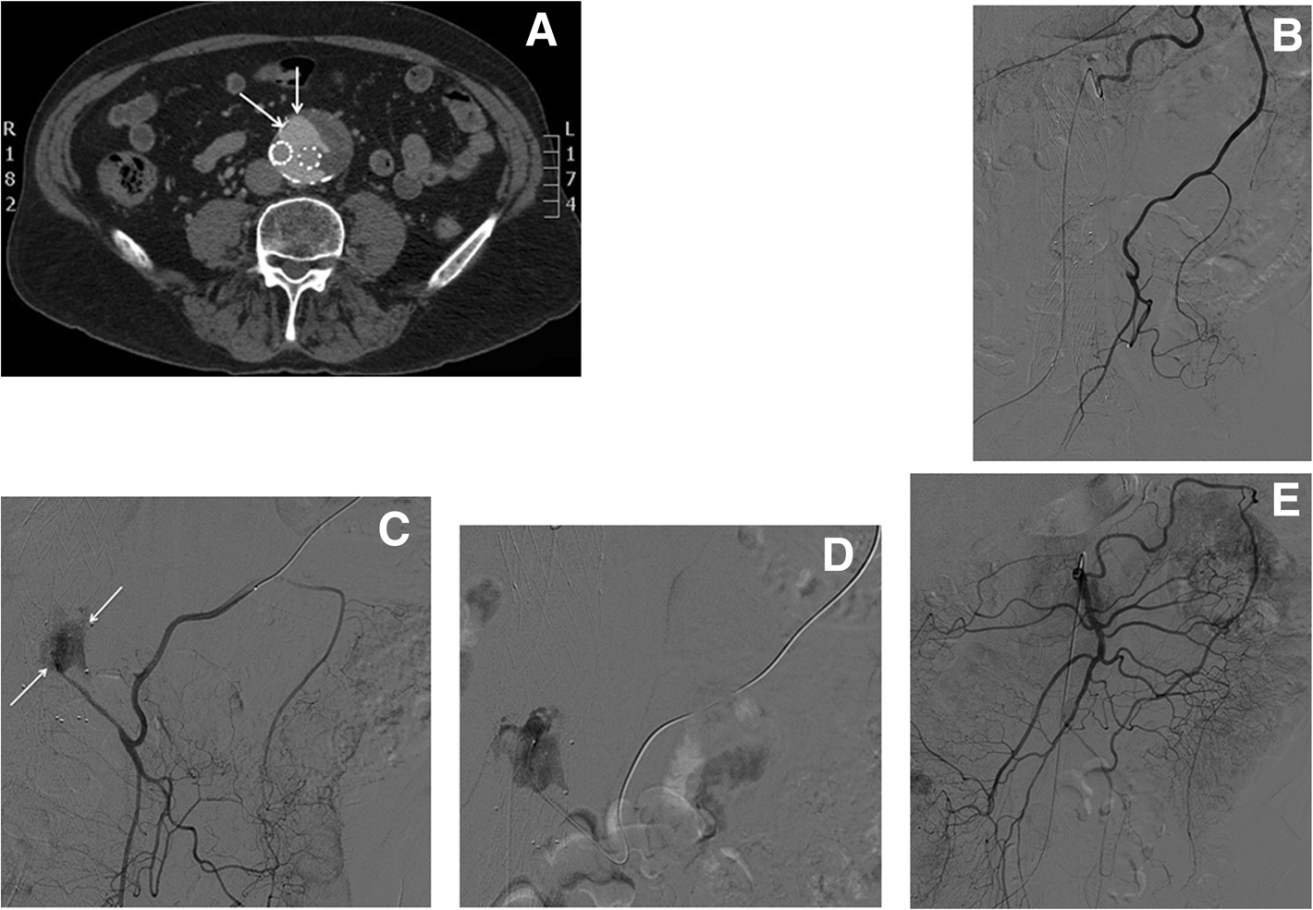
Type II endoleak embolization. **a** Contrast-enhanced CT in arterial phase shows the presence of a type II endoleak post EVAR (white arrows). **b** Transfemoral diagnostic DSA of the superior mesenteric artery shows hypertrophy of the Riolano arch. **c** Further diagnostic DSA performed by a coaxial microcatheter advanced in the Riolano arc confirms the endoleak opacification (white arrows) sustained by the inferior mesenteric artery stump. **d** Microcatheter advancement into the aneurysmatic sac and Squid embolization. **e** Final angiographic control shows complete endoleak exclusion

**Fig. 5:**
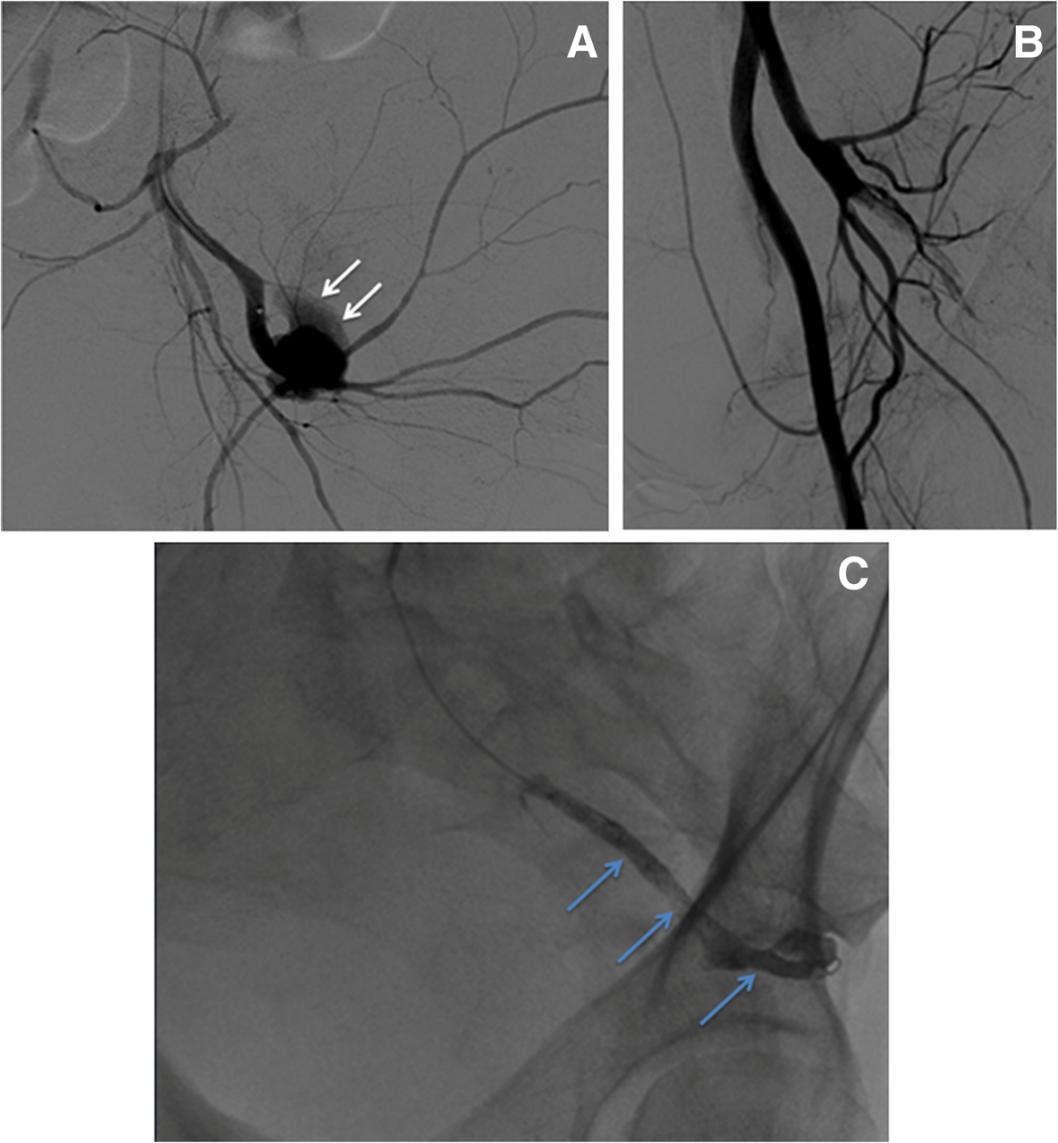
Iatrogenic acute arterial bleeding embolization. **a** Diagnostic DSA shows a significant contrast extravasation (white arrows) due to active arterial bleeding of a branch of the left hypogastric artery. **b** Progressive embolization with PVA particles and Squid-18. **c** After embolization, the bleeding vessel completely filled of Squid (light blue arrows)

**Fig. 6:**
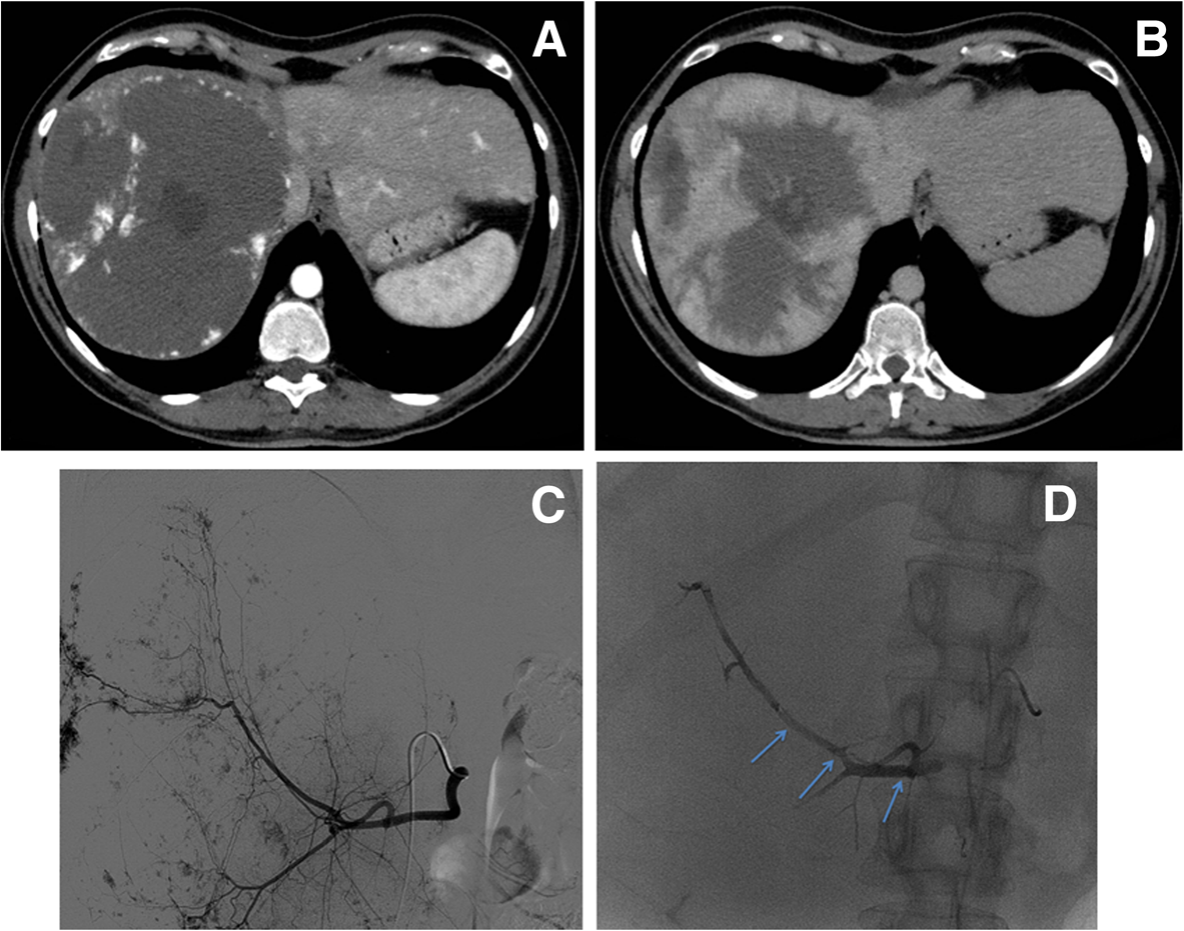
Preoperative embolization. **a** Contrast-enhanced CT in arterial phase of a hepatic giant hemangioma. **b** Contrast-enhanced CT in late phase. **c** Diagnostic DSA of the hepatic artery. **d** Final angiographic control after preoperative embolization using PVA particles and Squid (light blue arrows)

Thirty patients (16 females, 14 males, mean age = 59 years, age range = 24–88 years) affected by different abdominal diseases were submitted to embolization with Squid-Peri (Squid-18 in 15 cases, Squid-12 in 8 cases, Squid 18 + 12 in 7 cases) as follows: 10 cases of PVE (Fig. 1), 6 cases of VAAs (Fig. 2), 5 cases of AVMs (Fig. 3), 4 cases of type Type 2 endoleak (Fig. 4), 3 cases of pre-operative arterial embolization (Fig. 5), one case of female varicocele, and one case of emergency arterial bleeding following bone marrow biopsy (Fig. 6). Squid alone was used in 4 patients affected by type 2 endoleak, while in the remaining 26 patients it was used in combination with one or more than one other embolic agents as follows: PVA particles in 18 cases, coils in 13 cases, Amplatzer plugs in 3 cases. PVE were mainly performed using Squid and PVA particles, VAAs embolization using Squid and coils, AVMs with Squid and variable other embolic agents, while Amplatzer plugs were used in 3 different cases (VAA, AVM, preoperative embolization).

Embolization procedures were performed in election in 25 cases, in emergency in 5 cases (4 AVMs conditioning metrorrhagia in two cases, hematuria in one case, pulmonary embolism in one case, and 1 iatrogenic acute arterial bleeding following bone marrow biopsy).

Neither cases of microcatheter entrapment during Squid administration, nor cases of poor pain control during DMSO infusion were recorded.

All data regarding patients characteristics, abdominal disease, embolization type, Squid and other used embolic agents are reported in Table 1.

**Table 1:** Patients characteristics, abdominal disease, embolization type, Squid and other used embolic agents

Patient	Gender	Age	Symptomatic	Abdominal disease	Embolization type	Emergency/election	Squid type	Vials number	Other embolic agents
1	F	36	Yes (pulmonary embolism)	AVM (metastatic chorioncarcinoma)	Uterine arteries, gonadic vein	Emergency	18	1	PVA + Coils + Amplatzer Plug
2	F	38	Yes (metrorrhagia)	AVM	Uterine arteries	Emergency	18	1	PVA
3	M	77	No	Type II endoleak	Inferior mesenteric artery	Election	18	3	No
4	M	82	No	Type II endoleak	Inferior mesenteric artery	Election	18 + 12	1	No
5	M	34	Yes (pain)	AVM	Pelvic	Election	18	1	Coils + PVA
6	F	58	No	Hypervascular bone metastasis	Preoperative femoral artery	Election	18	1	Coils + PVA
7	F	41	No	Giant hepatic hemangioma	Preoperative hepatic artery	Election	18	1	PVA
8	F	55	No	VAA	Splenic artery	Election	18	2	Coils
9	F	62	No	VAA	Renal artery	Election	12	1	Coils
10	F	79	No	Colorectal liver metastases	PVE	Election	18	2	PVA + Coils
11	M	77	No	Cholangio-Ca	PVE	Election	12	3	PVA
12	M	74	No	Cholangio-Ca	PVE	Election	18	3	PVA
13	M	70	No	HCC	PVE	Election	12	3	PVA
14	M	79	No	HCC	PVE	Election	18	1	PVA + Coils
15	F	56	No	Colorectal liver metastases	PVE	Election	12	1	PVA
16	M	61	No	VAA	Splenic artery	Election	18	2	Coils
17	F	64	No	VAA	Splenic artery	Election	18	1	Coils
18	F	50	Yes (bleeding)	Arterial rupture	Left hypogastric artery	Emergency	18	1	Coils + PVA
19	M	55	No	Cholangio-Ca	PVE	Election	12	2	PVA
20	M	78	No	Colorectal liver metastases	PVE	Election	12	3	PVA
21	M	77	No	HCC	PVE	Election	18 + 12	2	PVA
22	F	56	No	VAA	Splenic artery	Election	18	1	Coils+Amplatzer Plug
23	M	27	Yes (dispnea)	Pulmonary sequestration	Preoperative bronchial artery	Election	18	1	Amplatzer Plug
24	M	88	No	Type II endoleak	Inferior mesenteric artery	Election	12	1	No
25	F	55	No	Cholangio-Ca	PVE	Election	12	1	PVA
26	M	77	No	VAA	Left gastric artery	Election	18	4	Coils
27	F	35	Yes (chronic pelvic pain)	Varicocele	Hypogastric veins	Election	18	2	Coils
28	M	78	No	Type II endoleak	Ileo-lumbar artery	Election	18	1	No
29	F	24	Yes (metrorrhagia)	AVM	Uterine arteries	Emergency	18	1	PVA
30	F	30	Yes (macrohematuria)	AVM	Renal artery	Emergency	18	1	PVA

### Primary and secondary outcomes

Technical success was achieved in 27/30 patients, with an overall technical success rate of 90%. Three patients (2 AVMs, 1 VAA) required re-intervention. After re-intervention technical success was achieved in all cases (100% of secondary technical success). 30-day clinical success was achieved in 28/30 patients (93.3%). Two patients submitted to PVE did not perform right hepatectomy due to inadequate FLR increase to the required cut off.

### Peri-procedural complications

Major peri-procedural complications were not recorded. Minor peri-procedural complications rate was 13.3%. Two asymptomatic patients 24 h after splenic artery aneurysm embolization showed a slight elevation of amylase levels (peak in one patient of 793 U/L and 321 U/L, respectively), without signs of pancreatitis at imaging: they were managed conservatively and in both cases, this pancreatic scree spontaneously resolved with prolonged hospitalization of one night (3 nights instead of 2 after the procedure). One of those 2 patients with distal position of the splenic aneurysm had also a distal ischemia (less than 10% of the spleen), without perisplenic fluid and with no clinical consequences. Another patient had a local hematoma spontaneously resolved due to the femoral catheterization.

### Squid administration modality

Two types of Squid were used: Squid-18 and Squid-12, characterized by high and low viscosity, respectively. Squid was mainly used in combination with other embolic agents (coils, PVA particles, and Amplatzer plugs) or alone (type II endoleak) to increase the embolizing power. Immediately prior to use, Squid was continuously mixed with a “shaker machine” (time = 20 min) to create a suspension of tantalum fluoroscopically visualizable. Squid was always administered using DMSO-compatible microcatheters: before Squid infusion, microcatheter dead space was filled with DMSO. Two types of microcatheteres were used: Renegade Hi-Flo (Boston Scientific, Natick, MA, USA), characterized by straight tip, length 135/150 cm, 0.027 in. internal lumen and Carnelian 2.2 (Tokai, Medical Products, Sarayashiki Taraga Kasugay-city, Japan) characterized by angled or cobra-shaped tip, length 135/150 cm, 0.021 in. internal lumen. The Squid was initially injected for 45 s to substitute the DMSO in the dead space of microcatheter and then into the target vessel, progressively retrieving the microcatheter to avoid its trapping, using a 1-ml syringe. The Squid was more slowly injected (60–90 s) to minimize DMSO toxicity and to ensure its tolerability also in case of TE of small vessels.

### Procedural data

Right PVE was performed through a right intercostal approach using a combined ultrasonography- and fluoroscopy-guided technique to access the portal vein (Venturini et al., 2005). A peripheral right portal vein was punctured with a Chiba 21G needle under ultrasonography guidance and then the main trunk of portal vein was catheterized with a standard 4-French catheter. After a preliminary portography, right PVE was performed after selective catheterization of the segments V-VI-VII-VIII using poly vinyl alcohol (PVA) particles (Boston Scientific, Natick, MA, USA), Squid (18 and 12) and coils in particular cases. If indicated, PVE was extended to IV and I segment.

All arterial procedures of transcatheter embolization (VAAs, AVMs, type 2 endoleaks, pre-operative embolization, acute arterial bleeding) were performed using a transfemoral approach (transaxillary in one case). After local anaesthesia, when necessary, a diagnostic aortography through a pig-tail catheter (4–5 French) was performed. A selective catheterization with standard 4-French angiographic catheters, such as Simmons or Cobra (Cordis, Miami, FL, USA), of the target artery was performed.

Different strategies of transcatheter embolization tailored for the specific disease were used.

For VAAs, initially the efferent vessels, then the aneurysm sac and finally the afferent vessel were embolized, progressively retracting the catheter or microcatheter according to the “isolation technique” (Ikeda et al., 2010; Venturini et al., 2017). VAAs embolization was performed using Squid, pushable (MReye, Cook, Bloomington, IN, USA) or detachable coils (Interlock, Boston Scientific, Natick, MA, USA) of variable diameter and length, and in particular cases, also Amplatzer Vascular Plugs (St Jude Medical, St Paul, MN, USA). The target artery and the nidus of AVMs were embolized using PVA particles, Squid, and in particular cases also coils. One Amplatzer plug was used only in one case of a giant AVM needing a simultaneous transfemoral arterial and transjugular approach: the plug was placed to occlude the right gonadic vein to simultaneously reduce the AVM outflow and to avoid further episodes of pulmonary embolisms (metastatic chorioncarcinoma conditioning giant pelvic AVM, pulmonary metastases and embolisms). Type 2 endoleaks were embolized using Squid alone after catheterization of the superior mesenteric artery or the hypogastric artery with standard 4-French angiographic catheters (Cobra, Simmons) and subsequent advancement of a coaxial microcatheter through Riolano arch-inferior mesenteric artery or ileo-lumbar artery, respectively. Preoperative embolization of the target artery was performed usually 1 day before surgery in case of giant masses or hypervascular tumors or pulmonary sequestration (Avsenik et al., 2015) using Squid and PVA (Amplatzer plug) to minimize the bleeding risk during surgery. Squid was used as embolic agent with PVA in case of acute arterial bleeding due to traumatic or iatrogenic large arterial rupture. Finally Squid was also used with coils using a transjugular approach to embolize distal branches of hypogastric veins in case of female varicocele conditioning a pelvic congestion syndrome after a preliminary phlebography performed with 4-French standard angiographic catheters (Headhunter, Cobra).

### PVE subgroup findings

Ten patients affected by cholangiocarcinoma (*n* = 4), colorectal liver metastases (*n* = 3) and HCC (*N* = 3) candidates to right hepatectomy were submitted to right PVE (segments V-VI-VII-VIII): 4 patients performed right PVE + IV segment, 2 patients right PVE + IV and I segment. 3 patients were also submitted to external biliary drainage before PVE due to obstructive jaundice. Technical success of PVE was 100%, while clinical success was 80%: after PVE, 2 patients were not submitted to right hepatectomy for insufficient growth of FLR-V demonstrated at contrast-enhanced CT performed 1 month after the procedure. .

The future liver remnant-volume (FLR-V) increased before PVE and after 4 weeks from 433.57 mL (range 245–647.8 mL) to 648.89 mL (range 371–1099.9 mL). (*P* value = 0,019) The mean increase of the future liver remnant-volume (FLR-V) induced by PVE was 53.55% (range 17.32–89.72%). After PVE, the %FLR/TELV increased from 30.24% to 43.75%, with a median increase of 48.29% (range 26.48–105.34%, P value = 0.015). The mean increase of TL-F after PVE at Dynamic ^99m^Tc-mebrofenin hepatobiliary scintigraphy (HBS) was 5,23%/min/m2 (range 2.2–6.92%/min/m2); the mean increase of FLR-F after PVE was 2.69%/min/m2 (range 0.44–4.95%/min/m2).

All volumetric and clinical data are reported in Table 2.

**Table 2:** Portal vein embolization (PVE): outcome measures

Patients	Gender	Age	Disease	Segments	FLR-V pre (mL)	FLR-V post (mL)	Increase FLR-V %	TELV pre (Ml)	TELV post (mL)	Embolized lobe-V pre (mL)	Embolized lobe-V post (mL)	FLR-V/ TELV pre (%)	FLR-V/ TELV post (%)	Increase FLR-V/TELV (%)	ASTpre (UI/L)	ALTpre (UI/L)	Bilipre (mg/dL)	ASTpost (UI/L)	ALTpost (UI/L)	Bilipost (mg/dL)	TL-F (%/min/m^2^)	FLR-F (%/min/m^2^)
1	F	79	MTS	RPVE+IVb	273.4	518.7	89.72%	1331.6	1230.3	1058.2	711.6	20.53%	42.16%	105.34%	51	0.52	71	61	0.72	0.72	/	/
2	M	77	Cholangio	RPVE	405.8	647.9	59.66%	1462.4	1846.1	1056.6	1198.2	27.75%	35.10%	26.48%	28	29	1.15	30	40	0.74	/	/
3	M	74	Cholangio	RPVE	413.8	707.6	71.00%	1439	1520.4	1025.2	812.8	28.76%	46.54%	61.85%	165	171	7.42	66	78	3.72	/	/
4	F	70	MTS	RPVE+IV	245.5	371.2	51.20%	1230.6	1137.1	985.1	765.9	19.95%	32.64%	63.63%	262	111	1.55	201	55	4.52	2.2	1.55
5	M	79	HCC	RPVE	640.7	751.7	17.32%	1453.5	1313.7	812.8	562	44.08%	57.22%	29.81%	76	118	1.02	52	71	0.7	6.58	4.95
6	F	56	MTS	RPVE+IV	647.8	813	25.50%	1461.7	1332.8	813.9	519.8	44.32%	61.00%	37.64%	164	435	0.51	73	235	0.6	5.15	4.1
7	M	55	Cholangio	RPVE+I + IV	360.3	504.4	39.99%	1402.3	1340.1	1042	835.7	25.69%	37.64%	46.49%	21	52	0.56	16	32	0.25	5.96	2.52
8	M	78	MTS	RPVE+I + IV	325.6	464.5	42.66%	1374.1	1492.9	1048.5	1028.4	23.70%	31.11%	31.31%	41	66	0.4	26	27	0.9	5.47	0.44
9	M	77	HCC	RPVE	675.4	1099.9	62.85%	1556.5	1857.1	881.1	757.2	43.39%	59.23%	36.49%	92	44	0.75	106	52	1.07	4.39	2.07
10	F	55	Cholangio	RPVE+IV	356.4	610	71.16%	1471.1	1750	1114.7	1140	24.23%	34.86%	43.88%	94	77	1.32	23	15	10.74	6.92	3.23

*ALT* Alanino Aminotransferase, *AST* Aspartate Aminotransferase, Bili bilirubine, *CRC-MTS* colo-rectal cancer metastases, *HCC* hepatocellular carcinoma, *FLR-V* future liver remnant volume, *FLR-F* future liver remnant function, *TELV* total estimated liver volume, *TL-F* total liver function

## Discussion

The choice to use a liquid embolic agent mainly combined with other agents (coils) for TE in various abdominal diseases was made to maximize the embolizing power with a good level of safety, owing to the poor tendency of the polymerized agent to migrate towards off target sites. Squid is a non-adhesive liquid embolic agent well known in cerebral field but with poor application in abdominal district (Erbahceci et al., 2014). It is composed of ethylene vinyl alcohol (EVOH) copolymer dissolved in dimethyl sulfoxide (DMSO) and micronized tantalum powder for radiopacity. Differently from the other most known EVOH-based liquid embolic agent Onyx, Squid is available in different combinations, with Squid-12 characterized by low density and viscosity. Squid-18, such as in case of Onyx use, is suggested for initial plug formation, while the new formulation Squid-12 can improve vascular penetration and spread more distally. Another advantage of Squid over Onyx is related to the reduced percentage (30%) of tantalum which can improve vessels visualization during embolization and limit metallic artifacts at imaging follow-up (Gioppo et al., 2017), as also recently demonstrated (Pop et al., 2018). Squid was always administered using DMSO-compatible coaxial microcatheters as straight Renegade Hi-flo (Boston) or curved Carnelian (Tokai): the peculiar cobra-shaped tip of Carnelian was useful in selected cases of marked tortuosity of the target artery, as for example in VAA (splenic) or type 2 endoleak embolization. No technical problems, such as microcatheter entrapment, were recorded. In the present study on 30 patients affected by different abdominal diseases, both formulations of Squid were used. Squid-12 was largely used in right PVE to embolize also the medium-peripheral branches of the right portal vein, usually after PVA particles administration. Embolization with Squid-18 was preferred in VAAs and AVMs usually combined with coils and PVA particles, respectively. The use of Squid, in association with other embolic agents, does not represent a limitation but rather a valid option, which increases the embolizing power. For example, in 6 patients submitted to VAA embolization using Squid and coils the occlusion process of the target artery was very rapid. According to the isolation technique (Ikeda et al., 2010), VAA embolization was successfully performed embolizing subsequently the efferent vessel, the aneurysmatic sac and the afferent vessel: Squid-18 slowly injected through the microcatheter during its careful retraction was able to produce a stable plug into the target artery strengthening the embolizing action of the coils: coils provide the support, while Squid can fill the hollows between the coils. In 2 patients affected by splenic artery aneurysms, a transient amylases increase after the procedure, conditioning a prolonged hospitalization of 24 h but spontaneously resolved, was recorded. It was probably caused by an accidental infusion of DSMO, differently from Squid not radiopaque and thus undetectable on fluoroscopy, in small pancreatic branches arising from the splenic artery. Pancreatic branches are probably more sensitive than other arteries to the DMSO toxicity (Pamuk et al., 2005). DMSO-infusion, which precedes Squid administration, must be performed very slowly. In our series of 5 patients, AVMs embolization strategy was initially based on PVA particles to determine nidus or microcirculation hypoxia, followed by Squid-18 to occlude the feeding arteries. In AVMs, it is mandatory before Squid administration to embolize nidus using PVA particles or embosphere (Merit Medical, Jordan, Utah, USA): the lack of peripheral occlusion with proximal embolization alone can favor collaterals formation from other vessels predisposing to relapses and re-interventions (Conger et al., 2016). Furthermore, in the case of an acute iatrogenic arterial bleeding Squid was very useful in emergency to quickly and completely fill the ruptured artery that presented a large breach with significant extravasation of contrast. Squid alone was used in 4 cases of type II endoleak embolization: after the advancement of a coaxial microcatheter in the Riolano arch or in the ileo-lumbar artery and its placement in the endoleak sac, the progressive filling with Squid of the aneurysmatic sac was successfully performed and a permanent occlusion was obtained. In the past, type 2 endoleak embolization was performed using different embolic agents such as glue, coils, thrombin and Onyx, combined or alone (Abullarage et al., 2012; Ierardi et al., 2017; Khaja et al., 2014). The advantage of using an EVOH-based liquid embolic agent, as Onyx or Squid, is related to its capacity to fill completely the sac including inflow and outflow vessels, forming a more stable plug than that provided by coils (Ierardi et al., 2017). Another significant advantage of EVOH-based liquid embolic agents is their complete detachment from the microcatheter tip, not always verifiable after glue infusion. Usually in type 2 endoleak embolization, after the placement of a standard angiographic in the superior mesenteric (or hypogastric) artery, microcatheter is advanced for a long distance before reaching the endoleak sac. The risk of an accidental spread of embolizing material from the microcatheter tip after embolization during its long retraction for many tens of centimeters without the protection of the angiographic catheter is potentially higher using glue than Squid (or Onyx): an accidental embolization of branches of inferior mesenteric or ileo-lumbar arteries with consequent ischemia is possible and it was previously described using glue (Bailey et al., 2011). Considering the clinical outcome of the whole population, our findings were globally satisfactory in terms of technical success, clinical success, re-intervention and recorded complications. In our heterogeneous group of patients, the largest subgroup of patients (*n* = 10) was that submitted to right PVE, extended to IV and I segments in selected cases, to reduce the risk of postoperative liver failure after right hepatectomy (Loffroy et al., 2015). In the past, different embolic agents were used for PVE, even though cyanoacrylate glue is usually considered the ideal embolic agent with the highest expected rate of liver regeneration (Jaberi et al., 2016). Onyx was previously used in PVE either in humans (Né et al., 2018) or in experimental models (Smits et al., 2012). In our series, Squid and PVA particles (coils in some cases) were used as embolic agents in PVE obtaining good outcomes in terms of technical success (100%), clinical success (80%), and also future liver remnant volume increase (more than 50%). In only two patients future liver remnant volume increase was not sufficient to perform right hepatectomy. 99mTc-mebrofenin hepatobiliary scintigraphy (HBS) associated to CT volumetry was useful to completely evaluate the function of the future liver remnant before surgery: in previous studies (de Graaf et al., 2010), it was particularly helpful in case of compromised liver (previous chemotherapy, cirrhosis). Summarizing in PVE, further studies with a larger cohort of patients and randomized trials will be necessary to establish if Squid and glue could be comparable in terms of liver regeneration induction.

Limitations of the present study are represented by the heterogeneity of abdominal diseases included, the simultaneous use of Squid with other embolic agents, the small sample size and the relatively short follow-up period.

## Conclusion

In this preliminary experience, Squid was successfully used with a low complication rate in many abdominal diseases, showing a valid emboliing action either combined with other embolic agents or alone. The availability of the formulationSquid-12 allows a better vascular penetration and the reduced tantalum concentration limits metallic artifacts at imaging post embolization compared to Onyx. However further studies with a larger cohort of patients will define the role of Squid as embolic agent in each specific abdominal disease.

## Abbreviations

AVMs: Arteriovenous malformations
BSA: Body surface area
CDU: Color Doppler Ultrasound
CT: Computed Tomography
DMSO: Dimethyl sulfoxide
DSA: Digital subtraction angiography
EVAR: Endovascular Aortic Repair
EVOH: Ethylene-vinyl alcohol copolymer
FLR: Future Liver Remnant
FLR-V: future liver remnant-volume
HBS: 99mTc-mebrofenin hepatobiliary scintigraphy
MRI: Magnetic Resonance Imaging
PVA: Poly vinyl alcohol
PVE: Portal vein embolization
TE: Transcatheter embolization
TELV: Total estimated liver volume
TL-F: Total liver-function
VAAs: Visceral artery aneurysms

## References

[CR1] Abularrage CJ, Patel VI, Conrad MF, Schneider EB, Cambria RP, Kwolek CJ (2012). Improved results using Onyx glue for the treatment of persistent type 2 endoleak after endovascular aneurysm repair. J Vasc Surg.

[CR2] Akmangit I, Daglioglu E, Kaya T, Alagoz F, Sahinoglu M, Peker A (2014). Preliminary experience with squid: a new liquid embolizing agent for AVM, AV fistulas and tumors. Turk Neurosurg.

[CR3] Alturki AY, Enriquez-Marulanda A, Schmalz P, Ogilvy CS, Thomas AJ (2018). Transarterial Onyx embolization of bilateral transverse-sigmoid Dural arteriovenous malformation with Transvenous balloon assist-initial U.S. experience with Copernic RC venous remodeling balloon. World Neurosurg.

[CR4] Angle JF, Siddigi NH, Wallace MJ, Kundu S, Stokes L, Wojak JC, Cardella JF (2010). Quality improvement guidelines for percutaneous transcatheter embolization: Society of Interventional Radiology standards of practice committee. J Vasc Interv Radiol.

[CR5] Avsenik J, Štupnik T, Popovič P (2015). Endovascular embolization prior to surgical resection of symptomatic intralobar pulmonary sequestration in an adult. Eur J Radiol Open.

[CR6] Bailey MA, McPherson SJ, Troxler MA, Peach AH, Patel JV, Scott DJ (2011). Ischemic skin ulceration complicating glue embolization of type II endoleak after endovascular aneurysm repair. J Vasc Interv Radiol.

[CR7] Brassel F, Meila D (2015). Evolution of embolic agents in interventional neuroradiology. Clin Neuroradiol.

[CR8] Chevallier O, Gehin S, Foahom-Kamwa A, Pottecher P, Favelier S, Loffroy R (2016). Ethylene-vinyl alcohol copolymer (Onyx®) transarterial embolization for post-traumatic high flow priapism. Quant Imaging Med Surg.

[CR9] Cobb RJ, Patterson B, Karthikesalingam A, Morgan R, Thompson M, Loftus I (2014). Onyx: a novel solution for a mycotic aneurysm. Cardiovasc Intervent Radiol.

[CR10] Conger JR, Ding D, Raper DM, Starke RM, Durst CR, Liu KC (2016). Preoperative embolizationof cerebral arteriovenous malformations with silk suture and particles: technical considerations and outcomes. J Cerebrovasc Endovasc Neurosurg.

[CR11] de Graaf W, van Lienden KP, Dinant S, Roelofs JJ, Busch OR, Gouma DJ (2010). Assessment of future remnant liver function using hepatobiliary scintigraphy in patients undergoing major liver resection. J Gastrointest Surg.

[CR12] Erbahceci Salik A, Islim F, Akgul A, Cil BE (2014). Concomitant transarterial and transvenous embolization of a pelvic arteriovenous malformation using a new liquid embolic agent, squid-12 and detachable coils. Case Rep Vasc Med.

[CR13] Gemmete JJ, Ansari SA, Gandhi DM (2009). Endovascular techniques for treatment of carotid-cavernous fistula. J Neuro Opthalmol.

[CR14] Gioppo A, Faragò G, Caldiera V, Caputi L, Cusin A, Ciceri E (2017). Medial Tentorial Dural Arteriovenous Fistula Embolization: Single Experience with Embolic Liquid Polymer SQUID and Review of the Literature. World Neurosurg.

[CR15] Ierardi AM, Franchin M, Fontana F, Piffaretti G, Crippa M, Angileri SA (2018). The role of ethylene-vinyl alcohol copolymer in association with other embolic agents for the percutaneous and endovascular treatment of type Ia endoleak. Radiol Med.

[CR16] Ierardi AM, Micieli C, Angileri SA, Rivolta N, Piffaretti G, Tonolini M (2017). Ethylene-vinyl alcohol copolymer as embolic agent for treatment of type II endoleak: our experience. Radiol Med.

[CR17] Ikeda O, Nakasone Y, Tamura Y, Yamashita Y (2010). Endovascular management of visceral artery pseudoaneurysms: transcatheter embolization using the isolation technique. Cardiovasc Interv Radiol.

[CR18] Izaaryene J, Vidal V, Bartoli JM, Gaubert JY (2016). Multiple bronchial artery aneurysms: successful treatment with ethylene-vinyl alcohol copolymer (Onyx®). Diagn Interv Imaging.

[CR19] Jaberi A, Toor SS, Rajan DK, Mironov O, Kachura JR, Cleary SP (2016). Comparison of clinical outcomes following glue versus polyvinyl alcohol portal vein embolization for hypertrophy of the future liver remnant prior to right hepatectomy. J Vasc Interv Radiol.

[CR20] Khaja MS, Park AW, Swee W, Evans AJ, Fritz Angle J (2014). Treatment of type II endoleak using Onyx with long-term imaging follow-up. Cardiovasc Intervent Radiol.

[CR21] Kim PH, Tsauo J, Shin JH, Yun SC (2017). Transcatheter arterial embolization of gastrointestinal bleeding with N-butyl cyanoacrylate: a systematic review and meta-analysis of safety and efficacy. J Vasc Interv Radiol.

[CR22] Laborda A, Medrano J, de Blas I, Urtiaga I, Carnevale FC, de Gregorio MA (2013). Endovascular treatment of pelvic congestion syndrome: visual analog scale (VAS) long-term follow-up clinical evaluation in 202 patients. Cardiovasc Intervent Radiol.

[CR23] Lau V, Sun M, Chu F (2013). Embolisation of hypervascular bone tumours: a pictorial essay with literature review. J Med Imaging Radiat Oncol.

[CR24] Loffroy R, Favelier S, Chevallier O, Estivalet L, Genson PY, Pottecher P (2015). Preoperative portal vein embolization in liver cancer: indications, techniques and outcomes. Quant Imaging Med Surg.

[CR25] Lv X, Song C, He H, Jiang C, Li Y (2017). Transvenous retrograde AVM embolization: indications, techniques, complications and outcomes. Interv Neuroradiol.

[CR26] Marcelin C, Le Bras Y, Petitpierre F, Midy D, Ducasse E, Grenier N, Cornelis F (2017). Safety and efficacy of embolization using Onyx® of persistent type II endoleaks after abdominal endovascular aneurysm repair. Diagn Interv Imaging.

[CR27] Né R, Chevallier O, Falvo N, Facy O, Berthod PE, Galland C et al (2018) Embolization with ethylene vinyl alcohol copolymer (Onyx®) for peripheral hemostatic and non-hemostatic applications: a feasibility and safety study. Quant Imaging Med Surg 8:280–290 10.21037/qims.2018.04.03 PMC5941214 29774181

[CR28] Nerva JD, Barber J, Levitt MR, Rockhill JK, Hallam DK, Ghodke BV (2018). Onyx embolization prior to stereotactic radiosurgery for brain arteriovenous malformations: a single-center treatment algorithm. J Neurointerv Surg.

[CR29] Olthof PB, Coelen RJS, Bennink RJ, Heger M, Lam MF, Besselink MG (2017). 99mTc-mebrofenin hepatobiliary scintigraphy predicts liver failure following major liver resection for perihilar cholangiocarcinoma. HPB (Oxford).

[CR30] Pamuk AG, Saatci I, Cekirge HS, Aypar U (2005). A contribution to the controversy over dimethyl sulfoxide toxicity: anesthesia monitoring results in patients treated with Onyx embolization for intracranial aneurysms. Neuroradiology.

[CR31] Pop R, Mertz L, Ilyes A, Mihoc D, Richter JS, Manisor M et al (2018) Beam hardening artifacts of liquid embolic agents: comparison between Squid and Onyx. J Neurointerv Surg. 10.1136/neurintsurg-2018-014542 10.1136/neurintsurg-2018-014542 30567844

[CR32] Rahimi S, Nassiri N, Huntress L, Crystal D, Thomas J, Shafritz R (2018). An institution-wide algorithm for treatment of type II Endoleak following endovascular aneurysm repair (EVAR). Vasc Endovasc Surg.

[CR33] Regine R, Palmieri F, De Siero M, Rescigno A, Sica V, Cantarela R, Villari V (2015). Embolization of traumatic and non-traumatic peripheral vascular lesions with Onyx. Interv Med Appl Sci.

[CR34] Ribé L, Bicknell CD, Gibbs RG, Burfitt N, Jenkins MP, Cheshire N, Hamady M (2017). Long-term results of intra-arterial onyx injection for type II endoleaks following endovascular aneurysm repair. Vascular.

[CR35] Sadeh-Gonike U, Magand N, Armoiry X, Riva R, Labeyrie PE, Lamy B (2018). Transarterial Onyx embolization of intracranial Dural fistulas: a prospective cohort, systematic review, and meta-analysis. Neurosurgery.

[CR36] Sakuhara Y, Nishio S, Morita K, Abo D, Hasegawa Y, Yuasa N (2015). Transcatheter arterial embolization with ethanol injection in symptomatic patients with enlarged polycystic kidneys. Radiology.

[CR37] Sheth RA, Sabir S, Krishnamurthy S, Avery RK, Zhang YS, Khademhosseini A, Oklu R (2017) Endovascular Embolization by Transcatheter Delivery of Particles: Past, Present, and Future. J Funct Biomater 8(2). 10.3390/jfb8020012 10.3390/jfb8020012 PMC5491993 28368345

[CR38] Shinkawa H, Takemura S, Tanaka S, Kubo S (2017). Portal vein embolization: history and current indications. Visc Med.

[CR39] Singfer U, Hemelsoet D, Vanlangenhove P, Martens F, Verbeke L, Van Roost D, Defreyne L (2017). Unruptured brain arteriovenous malformations: primary ONYX embolization in ARUBA (a randomized trial of Unruptured brain arteriovenous malformations)-eligible patients. Stroke.

[CR40] Smits ML, Vanlangenhove P, Sturm EJ, van den Bosch MA, Hav M, Praet M (2012). Transsinusoidal portal vein embolization with ethylene vinyl alcohol copolymer (Onyx): a feasibility study in pigs. Cardiovasc Intervent Radiol.

[CR41] Szatmáry Z, Hillman J, Finitsis S (2017). Meningioma embolization with the pressure cooker technique using squid 12. Interv Neuroradiol.

[CR42] van Lienden KP, van den Esschert JW, de Graaf W, Bipat S, Lameris JS, van Gulik TM, van Delden OM (2013). Portal vein embolization before liver resection: a systematic review. Cardiovasc Intervent Radiol.

[CR43] Venturini M, Angeli E, Maffi P, Fiorina P, Bertuzzi F, Salvioni M (2005). Technique, complications, and therapeutic efficacy of percutaneous transplantation of human pancreatic islet cells in type 1 diabetes: the role of US. Radiology.

[CR44] Venturini M, Marra P, Colombo M, Alparone M, Agostini G, Bertoglio L (2017). Endovascular treatment of visceral artery aneurysms and Pseudoaneurysms in 100 patients: covered stenting vs Transcatheter embolization. J Endovasc Ther.

[CR45] Venturini M, Marra P, Colombo M, Panzeri M, Gusmini S, Sallemi C (2018). Endovascular repair of 40 visceral artery aneurysms and Pseudoaneurysms with the Viabahn stent-graft: technical aspects, clinical outcome and mid-term patency. Cardiovasc Intervent Radiol.

[CR46] Yoon DJ, Jones M, Taani JA, Buhimschi C, Dowell JD (2016). A systematic review of acquired uterine arteriovenous malformations: pathophysiology, diagnosis, and Transcatheter treatment. AJP Rep.

